# Comparative Analysis of Regulatory Elements between *Escherichia coli* and *Klebsiella pneumoniae* by Genome-Wide Transcription Start Site Profiling

**DOI:** 10.1371/journal.pgen.1002867

**Published:** 2012-08-09

**Authors:** Donghyuk Kim, Jay Sung-Joong Hong, Yu Qiu, Harish Nagarajan, Joo-Hyun Seo, Byung-Kwan Cho, Shih-Feng Tsai, Bernhard Ø. Palsson

**Affiliations:** 1 Department of Bioengineering, University of California San Diego, La Jolla, California, United States of America; 2 Division of Molecular and Genomic Medicine, National Health Research Institutes, Miaoli, Taiwan; Uppsala University, Sweden

## Abstract

Genome-wide transcription start site (TSS) profiles of the enterobacteria *Escherichia coli* and *Klebsiella pneumoniae* were experimentally determined through modified 5′ RACE followed by deep sequencing of intact primary mRNA. This identified 3,746 and 3,143 TSSs for *E. coli* and *K. pneumoniae*, respectively. Experimentally determined TSSs were then used to define promoter regions and 5′ UTRs upstream of coding genes. Comparative analysis of these regulatory elements revealed the use of multiple TSSs, identical sequence motifs of promoter and Shine-Dalgarno sequence, reflecting conserved gene expression apparatuses between the two species. In both species, over 70% of primary transcripts were expressed from operons having orthologous genes during exponential growth. However, expressed orthologous genes in *E. coli* and *K. pneumoniae* showed a strikingly different organization of upstream regulatory regions with only 20% identical promoters with TSSs in both species. Over 40% of promoters had TSSs identified in only one species, despite conserved promoter sequences existing in the other species. 662 conserved promoters having TSSs in both species resulted in the same number of comparable 5′ UTR pairs, and that regulatory element was found to be the most variant region in sequence among promoter, 5′ UTR, and ORF. In *K. pneumoniae*, 48 sRNAs were predicted and 36 of them were expressed during exponential growth. Among them, 34 orthologous sRNAs between two species were analyzed in depth, and the analysis showed that many sRNAs of *K. pneumoniae*, including pleiotropic sRNAs such as *rprA*, *arcZ,* and *sgrS*, may work in the same way as in *E. coli*. These results reveal a new dimension of comparative genomics such that a comparison of two genomes needs to be comprehensive over all levels of genome organization.

## Author Summary

In order to investigate similarities and differences of closely related species, most of the comparative genomics studies focus on comparing the gene contents either shared or specific for each genome. However, it is also important to investigate the differences in non-coding regulatory elements because they influence the transcriptional and post-transcriptional processes. Thus, we performed a genome-wide profiling of transcription start sites (TSSs) in two species, *E. coli* K-12 MG1655 and *K. pneumoniae* MGH78578. Experimental identification of TSSs is important for precise definition of promoter regions and 5′ untranslated regions upstream of coding genes. Comparative analysis of these regulatory elements revealed the use of multiple TSSs, identical sequence motifs of promoter and Shine-Dalgarno sequence. However, we observed that the upstream regulatory regions of the majority of operons having orthologous genes were organized with different usage of promoters and TSSs, resulting in diverse and complex gene regulation. We also found that the 5′ UTR is the least conserved regulatory element in sequence between the two species. Moreover, 34 orthologous sRNAs between *E. coli* and *K. pneumoniae* were analyzed in depth. The analysis suggested many of *K. pneumoniae* sRNAs might regulate the target genes as in *E. coli*.

## Introduction

*Escherichia coli* K-12 MG1655 and *Klebsiella pneumoniae* MGH78578 belong to the same enteric family of bacteria of the class gammaproteobacteria. While *E. coli* K-12 MG1655 represents an extensively studied laboratory strain that is not known to be pathogenic, *K. pneumoniae* MGH78578 is a well-known pathogenic strain isolated from a patient with pneumonia [1]. There have been many comparative genomics approaches used to understand the similarities of closely related species in a wide range of genera such as *Escherichia*, *Klebsiella*, *Salmonella*, and *Listeria* [2], [3], [4], [5], [6]. These comparative genomics studies have mostly focused on comparing the gene contents, either shared or specific for each genome. However, it is also important to investigate the similarities and differences in non-coding regulatory elements including promoter, 5′ untranslated region (5′ UTR), and small RNA (sRNA), due to their influence on transcriptional and post-transcriptional processes.

The transcription start site (TSS) is where transcription begins and is the +1 position of the 5′ untranslated region (5′ UTR) of mRNA. The promoter, which governs the ability to initiate transcription and control the expression of genes, is directly upstream of the TSS. The identification of promoter elements in DNA by computational methods depends on the statistical analysis of consensus sequences as overrepresented regions [7], [8]. Regulatory sequence elements have been studied by computational methods based on the genomic sequence of the non-coding upstream region [9], [10], [11], however those sequence elements in promoters are short and not fully conserved in the sequence. Thus, there is a high probability of finding similar sequence elements outside the promoter regions. In the case of the TSS, the region is not overrepresented enough by any consensus sequences and is thus difficult to predict by computational efforts. However, when the TSS is known, the DNA region most likely to contain regulatory binding sites is circumscribed, and the effectiveness of searching sequence motifs of interest is greatly enhanced [12]. Thus, determining the precise locations of TSSs by experimental methods is necessary to accurately annotate the promoter region and the untranslated region. Knowledge of the 5′ UTR region is important for studying the sequence and structure of the 5′ end of mRNA (which is associated with transcription regulation, mRNA transcript stability, and translational efficiency) because translational efficiency in bacteria is often controlled by RNA-binding proteins, noncoding regulatory RNAs, endoRNases, the 30S subunit of ribosome, and structural rearrangements within 5′ UTR [13].

Genome-wide identification of TSSs with the aid of deep sequencing has allowed researchers to reveal a landscape of TSSs across the whole genome in many microorganisms, including *E. coli* [14], [15], [16], *H. pylori* [17], *G. sulfurreducens* [18], and other species [12], [19]. In these studies, experimental TSS datasets were used to understand the transcription architecture, to appreciate the complexity of genomic structure, and to analyze regulatory elements for each species. Comparison of regulatory elements, which can be addressed by experimentally determined TSSs under the same growth condition, is expected to elucidate any regulatory similarities or differences, based not only on the genomic sequence, but also on the transcriptional context of compared species as well.

Here, we carried out the genome-wide TSS profiling experiments for *E. coli* K-12 MG1655 and *K. pneumoniae* MGH78578 to accurately determine the boundaries in the regulatory regions between the promoter region and the 5′ UTR. The upstream regulatory regions between those two closely related species were then compared to investigate whether those regions are conserved and organized in similar manners. In addition, we used the TSS dataset to identify sRNAs in *K. pneumoniae*, because very little is known about them. We then compared the *K. pneumoniae* sRNAs to orthologous sRNAs in *E. coli*, in terms of sequence conservation and their target sites. The range of sequence conservation or diversion between non-coding regulatory elements in interspecies microorganisms could lead to insights about regulatory features that may also play similar roles in the respective species.

## Results

### Experimental identification of TSSs in *E. coli* and *K. pneumoniae*

Primary mRNA transcripts in prokaryotes are triphosphorylated at the 5′ ends. We isolated total RNA from *E. coli* and *K. pneumoniae* cells growing in mid-exponential phase, and enriched primary mRNAs by removing any monophosphorylated ribosomal 23S, 16S rRNA, tRNA, and any degraded mRNAs by treatment with terminator exonuclease [17], [18]. By using a modified 5′RACE (rapid amplification of cDNA ends) followed by deep sequencing as described in [18], libraries were prepared and sequenced to determine potential TSSs for each strain of *E. coli* K-12 MG1655 and *K. pneumoniae* MGH78578. These TSS libraries yielded >11.6 million and >2.4 million sequence reads for *E. coli* and *K. pneumoniae*, respectively. 15.70% and 19.60% of those sequence reads were uniquely mapped with 36 bp read length onto the *E. coli* and *K. pneumoniae* reference genomes respectively. Unique sequence reads that perfectly matched the respective genome sequence were mapped to annotate a total of 3,746 and 3,143 TSSs for the *E. coli* K-12 MG1655 and *K. pneumoniae* MGH78578 genome, respectively (Table S1). The average number of TSS reads of *E. coli* and *K. pneumoniae* TSSs was 107.8 and 78.5, respectively. The lower number of identified TSSs for *K. pneumoniae* could be due to a lesser number of sequence reads, and this factor was taken into account in further analysis.

To verify the quality of the TSS data, we compared our experimental *E. coli* TSS data with previously published *E. coli* TSS datasets [15], [16]. There is no public genome-wide TSS dataset available for *K. pneumoniae*, which is why only TSS data for *E. coli* was used for this analysis. In RegulonDB, there are 1258 upstream sense TSSs annotated for *E. coli*, generated by 5′ triphosphate enrichment method. 624 (49.6%) TSSs out of 1258 matched exactly with TSSs of this study, and 257 (20.4%) TSSs matched within 3 bp tolerance. Thus, 70.0% of known TSSs from RegulonDB agreed with the TSSs from our study. From the TSS dataset generated without 5′ triphosphate enrichment method, 3661 TSSs were reported for the exponential growth condition. 1603 (43.8%) TSSs matched exactly with TSSs of this study, and 527 (14.4%) TSSs matched within 3 bp tolerance. In sum, 58.2% of TSSs were found in TSSs of this study. A comparison of our TSS dataset with two other datasets suggested TSS datasets generated by a similar method were in better agreement, and *E. coli* TSSs determined by an independent experiment were matched by TSSs used in this study.

**Figure 1:**
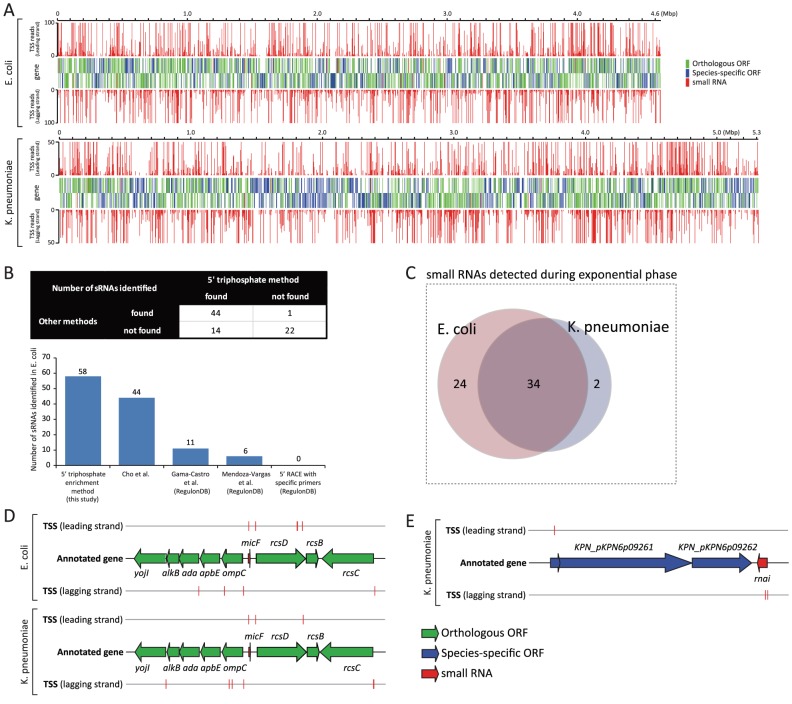
Experimentally determined TSSs and their association with annotated genes. (A) Genome-wide TSS mapped onto *E. coli* and *K. pneumoniae* genome annotation. (B) Number of *E. coli* sRNAs detected with 5 TSS datasets generated by different methods. (C) Number of sRNAs detected from *E. coli* and *K. pneumoniae* during the exponential growth. (D) Schematic drawing of annotated TSSs assigned to orthologous *micF* sRNA and coding genes surrounding *micF* in *E. coli* and *K. pneumoniae*. (E) Schematic drawing of annotated TSSs assigned to *K. pneumoniae* sRNA, *rnai*, and coding genes near *rnai*.

A genome-wide TSS landscape of *E. coli* and *K. pneumoniae* was built by assigning TSSs to the nearest downstream gene including ORFs and sRNAs, but excluding TSSs located beyond 700 bp from the translation start site of the closest ORF in a strand specific manner (Fig. 1A). In *E. coli*, TSSs were assigned to 2654 genes, while TSSs in *K. pneumoniae* were assigned to 2301 genes (2175 genes in the main chromosome, and 126 genes in the plasmids).

### Identification of small RNAs in *K. pneumoniae*

While over 80 sRNAs have been identified and experimentally verified in *E. coli*, very little is known about *K. pneumoniae* sRNAs. Identifying the occurrence of sRNAs and determining their boundaries in a genome-wide manner is challenging, especially for less studied organisms, because sRNAs generally have no clear-cut signatures unlike protein-coding genes, which are specified by a genetic code. In order to overcome limitations of previous experimental approaches, and to interrogate sRNAs in a genome-wide manner, a deep-sequencing approach was applied and proved successful [20]. Before investigating the possible presence of sRNAs in *K. pneumoniae*, *E. coli* TSS datasets were analyzed to assess how many currently annotated sRNAs in *E. coli* could be identified under the experimented condition, and how well TSS signals were matched with 5′ ends of those sRNAs. In addition, TSS datasets generated with the 5′ triphosphate enrichment method in this study were compared to four other TSS datasets generated by different methods [14], [15], [16] in the light of using 5′ triphosphate enrichment. Many sRNAs are subjected to post-transcriptional processing, however, which results in an accumulation of shorter products with 5′ monophosphate [21], [22], [23]. Therefore, only unprocessed sRNAs or precursor transcripts of sRNAs, which have 5′ triphosphate and can be detected by this method, were analyzed.

Of 81 annotated sRNAs in *E. coli*, 58 (71.6%) had corresponding TSSs, and were thus considered to be expressed during exponential growth. Expression profiling data taken from [16] also supported the expression of those sRNAs under the experimented condition, although *rprA* showed no significant expression according to that data (Figure S1). This could be because *E. coli* RprA transcript is subject to specific endoribonuclease cleavage [21], resulting in the accumulation of processed shorter form, which is not long enough to be detected by the tiling array. TSS signals were well matched with the 5′ ends of unprocessed or precursor transcripts of 58 sRNAs including *rprA* (Fig. 1B, Table S2). In comparison, TSS datasets generated by deep-sequencing without 5′ triphosphate enrichment [16] presented TSS signals for 44 sRNAs (54.3%). Three other TSS datasets, generated by other methods [14], [15], were obtained from RegulonDB database (http://regulondb.ccg.unam.mx/). They showed TSSs assigned to 11 (13.6%), 6 (7.4%), and 0 (0%) sRNAs for each method (Fig. 1B). Thus, experimental TSS generated by deep sequencing is a practical indicator that shows the occurrences of sRNAs in *E. coli* and determines the genomic positions of their 5′ ends. Additionally, our TSS dataset detected the largest number of annotated sRNAs in *E. coli*, compared to previous methods. We believe this result shows that the TSS dataset for *K. pneumoniae*, generated with the same method, can be used to detect possible sRNAs in that species and to determine the 5′ ends of those sRNA candidates.

In order to identify and confirm the occurrence of sRNAs in *K. pneumoniae* by experimentally determined TSSs, tentative sRNA candidates should first be predicted by computational methods. A number of computational algorithms have been developed over the last decade for the purpose of predicting sRNAs in bacterial genomes, and primary sequence conservation in closely related species is one of the most useful data types for predicting whether a genomic sequence corresponds to an sRNA [24]. Since a majority of *E. coli* annotated coding genes (63.7%) have homologs in the *K. pneumoniae* genome, and conserved sRNAs are frequently identified adjacent to conserved coding genes in other organisms, we looked up the closest orthologous ORFs to annotated sRNAs of *E. coli*, and then searched tentative sRNA sequences in *K. pneumoniae* genomic sequences bound to those neighboring orthologous genes. For example, in *E. coli*, *micF* sRNA is surrounded by *ompC* and *rcsD*, both of which are conserved coding genes between the two species. The *K. pneumoniae* genomic sequence bound to *ompC* and *rcsD* orthologous ORFs was used for searching the genomic sequence of *micF* by sequence alignment (Fig. 1D, detailed method described in Materials and Methods section). This approach was supplemented by running Infernal algorithm [25] with sRNA models from the Rfam database 10.1 (http://rfam.sanger.ac.uk/). Using this combined approach, we identified 48 tentative sRNAs in the *K. pneumoniae* genome, and 36 of them were expressed by associated TSSs (Fig. 1C, Table S3). Expression of those sRNAs was also supported with expression profiling data, with the one exception being *rprA* (Figure S1). *rprA* of *K. pneumoniae* showed no significant level of transcription according to the expression profiling data, however *rprA* had an assigned TSS with 1865 reads, which was also observed in *E. coli rprA* with an assigned TSS of 3012 reads. This indicates a possibility of post-transcriptional processing of *K. pneumoniae* RprA transcript as is the case in *E. coli*. 47 of 48 putative sRNAs were located in the main chromosome (NC_009648) of *K. pneumoniae*, while one sRNA, *rnai*, was found in the plasmid (NC_009652) (Fig. 1E).

Of 36 small RNAs detected during the exponential phase in *K. pneumoniae*, 34 had orthologous sRNAs in *E. coli*, leaving 2 non-orthologous sRNAs, *rnai*, and *ryhB-2* (Figure S1, Figure S2 and Figure S3A). Their expression was supported by TSS and expression profiling (Figure S3A). *ryhB-2* was so-named because another orthologous *ryhB* sRNA was identified in a position between orthologous ORFs *yhhX* and *yhhY*. *rnai* non-coding RNA is an antisense repressor of the replication of some *E. coli* plasmids [26]. While *E. coli* K-12 MG1655 does not have any plasmid, *K. pneumoniae* MGH78578 has 5 plasmids, one of which (NC_009652) contains *rnai* sRNA.

### Similar usage of regulatory features

**Figure 2:**
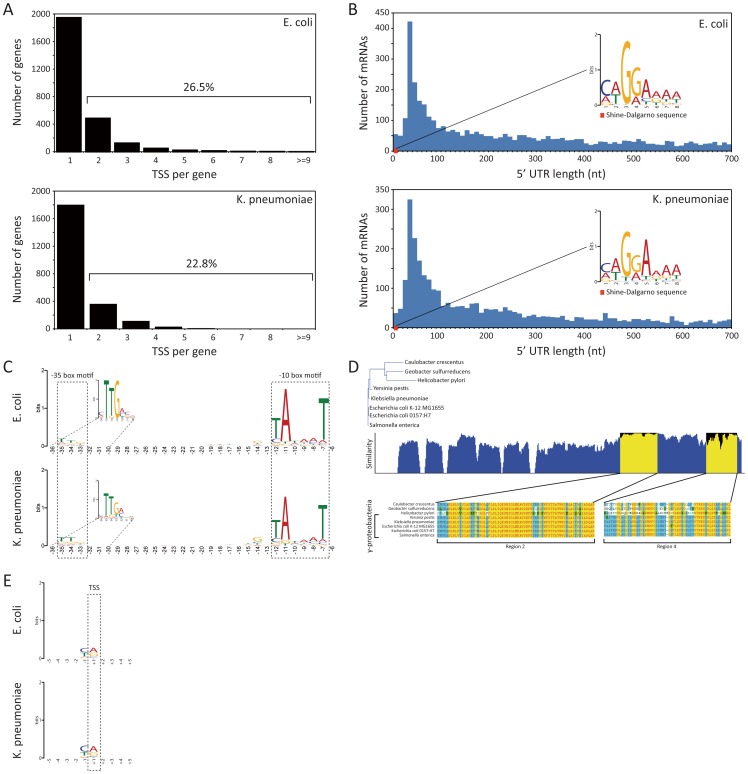
TSS annotation and structure of promoter region and 5′ UTR. (A) Number of TSSs assigned per annotated genes. (B) Distribution of 5′ UTR lengths for *E. coli* and *K. pneumoniae*, and the Shine-Dalgarno sequence motif. (C) Sequence motif of promoter region containing −10 and −35 boxes. (D) Conservation of RpoD amino acid sequences of 5 species in gammaproteobacteria and 3 other species belonging to proteobacteria. (E) Di-nucleotide preference near the TSS site.

The majority of *E. coli* annotated genes, 1945 (73.5%), were annotated with a single TSS, and the remaining 26.5% had multiple TSSs mainly ranging from 2 to 7, allowing alternative transcripts (Fig. 2A). Similar to the complex organization of promoter regions and usage of multiple TSSs shown in *E. coli*, 534 (22.8%) of *K. pneumoniae* annotated genes had multiple TSSs, leaving a large fraction of genes, 1802 (77.2%), which were assigned to a single TSS (Fig. 2A).

In order to investigate other regulatory features shared by *E. coli* and *K. pneumoniae*, the length distribution of the 5′ UTR bounded by experimental TSS and translational start site was calculated, and possible sequence motifs were examined with the MEME motif search algorithm [27]. The length of the 5′ UTR ranged from 0 to 700 nucleotides, with the most abundant length found to be between 25 to 35 bp for both bacterial species (Fig. 2B). For 18 genes from *E. coli* and 10 genes from *K. pneumoniae*, leaderless mRNAs with the TSSs corresponding exactly to the start codon were found. The leaderless mRNAs encoded proteins of various functions (Table S4).

Experimentally determined TSSs in *E. coli* and *K. pneumoniae* were used to detect the Shine-Dalgarno (SD) sequence of the ribosome binding site (RBS). Expecting to find that motif within the boundaries of the 5′ UTR, which are defined by the TSS and translation start site of the downstream ORF, we took sequences from 5′ UTR regions in *E. coli* and *K. pneumoniae* and searched for consensus motifs. A conserved caGGaaaa sequence motif (lower-case characters indicate an information content <1 bit) was found in *E. coli*, and an identical conserved caGGaaaa motif was also found in the 5′ UTR of *K. pneumoniae*. The most dominant distance between the SD sequence motif and translational start site was 6 nucleotides in both species. Motif logos for both species are illustrated in Fig. 2B.

Bacterial promoters usually contain specific sequences, which RNA polymerase-associated sigma factors can recognize and to which they can bind. For example, the *E. coli* housekeeping sigma factor σ^70^ (*rpoD*, b3067) is known to recognize −10 (TATAAT) and −35 (TTGACA) boxes [28]. Although sequence motifs of major *E. coli* sigma factors have been investigated by experimental and computational approaches, less is known for *K. pneumoniae* sigma factors and their binding motifs. *E. coli* and *K. pneumoniae* are closely related, and they share major sigma factors, such as *rpoD*, *rpoS*, *rpoH*, *rpoN*, and *rpoE* with a high level of amino acid sequence conservation over 95%, with the exception of *rpoN* that has 89.8% amino acid sequence similarity (Table S5). Since sigma factor σ^70^ is housekeeping during exponential growth in *E. coli* and presumably in other gammaproteobacteria including *K. pneumoniae* as well, conservation of subregions 2 and 4 of bacterial sigma factor σ^70^, which are known to recognize the −10 and −35 boxes, can give insights toward understanding the promoter structure of *K. pneumoniae*. Thus, amino acid sequences of *rpoD* of 5 strains belonging to gammaproteobacteria and 3 strains belonging to other classes were aligned and analyzed (Fig. 2D). Notably, region 2, which recognizes the −10 box, was perfectly conserved among species in gammaproteobacteria, and region 4, which recognizes the −35 box, was almost conserved as well. Since the conservation of sigma factor σ^70^ subregions recognizing sequence motifs in the promoter and the expression of housekeeping *rpoD* in *E. coli* and *K. pneumoniae* was confirmed with the TSS dataset and expression profile, it is likely that the promoter structure of those species are identical. Thus, TSSs in *E. coli* and *K. pneumoniae* identified in this study were used to find sequence motifs of the promoter region, which includes the −10 and −35 boxes, in order to see whether two closely related bacteria share similar or identical promoter sequence motifs. We extracted 50 bp long sequences directly upstream of the TSSs, which are long enough to cover the −10 and −35 boxes, and ran the MEME motif search algorithm. As a result, the consensus sequence of the extended Pribnow box motif (tgnTAtaaT) including the −10 box was obtained, and the −35 box sequence motif (cTTgaca) was also found, as expected (Fig. 2C). Moreover, the most dominant distances between the −10 box and TSS and between the −10 and −35 boxes were also the same in both bacteria. Although the sequence motif obtained herein is based on genome-wide TSS profiles generated only under exponential growth and other sigma factors having different binding sequence motifs may play a minor role in transcription regulation under the experimented condition, overrepresented sequence motifs of promoter regions in *E. coli* are in accordance with prior knowledge, and the two species in this study showed identical sequence motifs of the promoter. Thus, these closely related species seem to share identical promoter structures, reflecting a high conservation of major sigma factors.

Previous studies have shown evidence of a purine (A/G) preference at the TSS in *E. coli* [29]. Here, we investigated if the experimentally derived TSS data provide insights into any such nucleotide preference at the TSS. Thus, nucleotide preferences from −5 to +5 sites surrounding the TSSs for *E. coli* and *K. pneumoniae* were calculated. The current experimentally derived TSSs in both species also showed a significant dinucleotide preference at the +1 TSS and −1 site (Fig. 2E). In *E. coli*, 78.6% of the TSSs were represented by purine base (45.2% A and 33.4% G) at the TSS. Similarly, 79.4% of *K. pneumoniae* TSSs presented the purine base (48.0% A and 31.4% G) at that site. Interestingly, another nucleotide preference at the −1 site, the nucleotide before the TSS and the last nucleotide that is not transcribed, was observed in both species. In *E. coli*, 80.2% showed the pyrimidine base (35.4% T and 44.8% C) preference at the −1 site. Likewise, in *K. pneumoniae*, 81.5% of cases also showed the pyrimidine base (31.0% T and 50.5% C) at the −1 site. Flanking regions ranging from +2 to +5 sites and −2 to −5 sites showed no significant nucleotide preference (Fig. 2E). Thus, both species showed the purine preference at the +1 TSS and the pyrimidine preference at the −1 site. In accordance with this observation, *H. pylori*, which belongs to a different class of alphaproteobacteria, also showed purine preference at the TSS (66.0% A or G) and pyrimidine preference at the −1 site (68.3% T or C) [17]. Similar to the dinucleotide sequence preference at +1 and −1 sites found in bacteria, transcription from the *S. cerevisiae* promoter [30] and the mammalian [31] promoter preferentially starts with a purine at position +1, having a preference for pyrimidine at position −1.

These results suggest that *E. coli* and *K. pneumoniae* share many regulatory features at the transcriptional and translational level. They have a conserved promoter structure reflecting preserved sigma factors, use multiple TSSs that extensively increase transcriptome complexity by resulting in alternative transcripts, and show dinucleotide preference near the TSS position. In addition to this similarity in transcriptional features, *E. coli* and *K. pneumoniae* exhibit conserved Shine-Dalgarno sequence motifs, the same distance from Shine-Dalgarno motif to translation start site, and 5′ UTR length distribution, suggesting similarity in regulatory features of translation.

### Different organization of the upstream regulatory region

**Figure 3:**
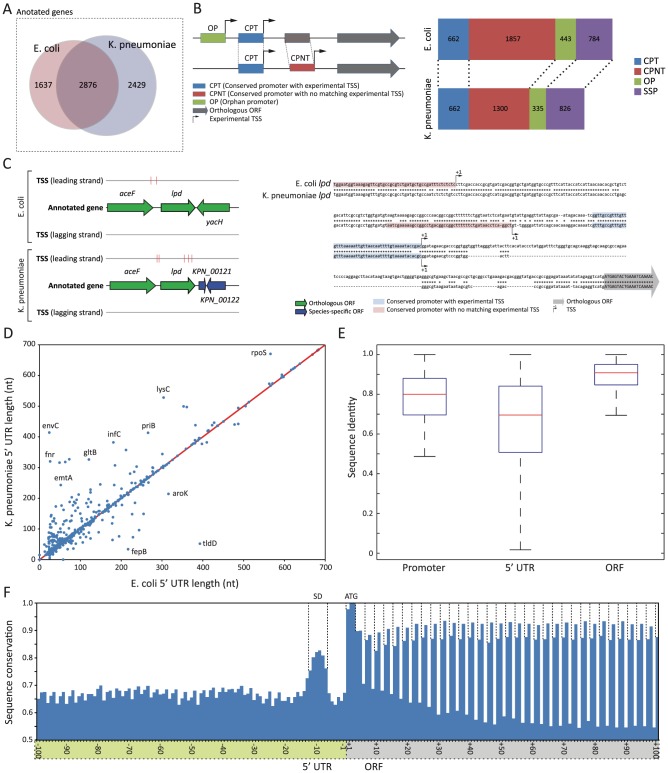
Different organization of upstream regulatory region between *E. coli* and *K. pneumoniae*. (A) Venn diagram showing orthologous genes and species-specific genes between *E. coli* and *K. pneumoniae*. (B) 4 different types of promoter regions, and their numbers identified in two species. (C) Schematic drawing of annotated TSSs and sequence comparison of regulatory region upstream of *lpd*. (D) Length difference between the pairs of comparable 5′ UTR. (E) Comparison of sequence conservation of promoter, 5′ UTR, and ORF regions. (F) Sequence conservation of genomic regions surrounding translation start sites.

While *E. coli* and *K. pneumoniae* share several regulatory features, it is still unknown whether the two species use them to regulate gene expression in the same manner. Thus, we analyzed the usage of regulatory elements upstream of orthologous genes between two strains in order to investigate whether those conserved genes are regulated in a similar or different manner. The orthologous genes present in *E. coli* and *K. pneumoniae* were selected by reciprocal alignments using a threshold of 50% amino acid sequence similarity and 50% alignment length between the encoded proteins, resulting in a set of 2,876 orthologs (Fig. 3A, Table S5). 2962 (79.1%) *E. coli* TSSs were assigned to orthologous genes defined herein, and in *K. pneumoniae*, 2317 (73.1%) of TSSs were assigned to orthologous genes. Considering 63.7% (2876 out of 4513) of genes in *E. coli* and 54.2% (2876 out of 5305) of genes in *K. pneumoniae* were orthologous, detection of over 79.1% of TSSs in *E. coli* and 73.1% in *K. pneumoniae* assigned to orthologous coding genes implies over 73% of primary transcripts were expressed from operons or transcription units having orthologous genes at the first position. In *E. coli*, the average number of genes in an operon is about 1.5 [16], and operons containing orthologous genes in *E. coli* have a tendency to keep their sequential position in *K. pneumoniae*, suggesting possible conservation of operon structures. This result suggests that the majority of primary transcripts were expressed from operons containing conserved orthologous genes during exponential growth in both species. Thus, further analysis of regulatory regions upstream of orthologous genes with genome-wide TSSs covers a majority of expressed gene contents under the experimented condition.

Despite the fact that orthologous genes were used to express the majority of primary transcripts during exponential growth, regulatory regions upstream of those conserved coding genes were organized in a different manner with multiple TSSs (Fig. 3B). In order to perform a detailed investigation comparing promoter regions between two species, each TSS was used to define a promoter region. A promoter region was defined as 50 bp long nucleotide sequences upstream of each TSS, which was long enough to include most of the regulatory elements identified, including the −1 site, −10 box, and −35 box, but not too long to exclude unnecessary sequences. Then, the promoter region was categorized into one of four groups, based on sequence conservation of the promoter region and presence of an experimental TSS: conserved promoter region with TSS (CPT), conserved promoter region with no matching TSS (CPNT), orphan promoter region (OP), or species-specific promoter (SSP). CPT was defined as a promoter region with a conserved sequence in both strains with a matching experimental TSS, and was used to define the promoter region and 5′ UTR, which were comparable between the two species. CPNT was defined as a promoter region with a conserved sequence in both strains, however with experimentally determined TSSs in only one species. Similarly, OP was defined as a promoter region with no conserved sequence between *E. coli* and *K. pneumoniae*, and with experimental TSSs in only one species. SSP was defined as a promoter region upstream of non-orthologous genes. (More details described in Materials and Methods section)

If the sequence of regulatory regions upstream of orthologous coding genes is also conserved, then conserved promoter (CPT) should be the most frequent type of promoter region. However, an exhaustive comparison of promoter regions resulted in only 662 conserved promoters (CPT) between *E. coli* and *K. pneumoniae*, which covered 17.7% of TSSs and corresponding promoter regions in *E. coli* and 21.1% in *K. pneumoniae*. An unexpectedly small portion of conserved promoter regions with matching TSSs in two species under the exponential growth supports a different organization of regulatory regions containing multiple TSSs and their associated promoters between those two closely related species. Interestingly, in both species, the promoter type with the largest number was the conserved promoter with no matching TSS (CNPT). In *E. coli*, 49.6% of TSSs were associated with promoters with conserved sequence, and no matching TSSs were found upstream of corresponding orthologous genes in *K. pneumoniae*. Similarly, 41.3% of TSSs of *K. pneumoniae* were associated with that type of promoter. A smaller number of TSSs was detected in *K. pneumoniae* versus *E. coli*, despite *K. pneumoniae* having the larger genome. This was possibly due to fewer raw reads being obtained from the *K. pneumoniae* TSS library. Thus, it is arguable that the portion of conserved promoters with matching TSSs could increase as the coverage of TSS reads goes up. However, over 40% of promoters with conserved sequences had TSSs in one species, but had no matching TSS in the other species. Thus, the regulatory regions upstream of orthologous genes are organized in a different manner, despite a large portion of promoters having conserved sequences between two species. This suggests different sets of TSSs are used to express those orthologous genes. For example, *lpd* had two experimental TSSs in *E. coli* and *K. pneumoniae* (Fig. 3C). Proximal TSSs were matching, and had a highly conserved promoter sequence. Distal TSSs of *lpd* had conserved sequences, but were in different locations. Moreover, promoters with no conserved sequence and TSS in one species (OP, orphan promoter) also support that interpretation. Thus, while two closely related species may share identical transcriptional machineries including sigma factors and RNA polymerase, upstream regulatory regions are organized differently, so that even conserved genes can be regulated differently, and in many cases mRNA transcripts from orthologous genes can have different 5′ UTRs, which may have disparate regulatory elements in that region.

To investigate similarities and differences in 5′ UTR regions, their length and sequences were defined by 662 comparable conserved promoter regions with TSSs in both species, as shown in Fig. 3D and Fig. 3E. The length comparison of the 5′ UTR between *E. coli* and *K. pneumoniae* showed a strong correlation (R^2^ value of 0.877), and 169 (25.5%) 5′ UTR regions had exactly the same length. However, in general, the *K. pneumoniae* 5′ UTR was longer than that of *E. coli*, reflecting the bigger size of the genome (Fig. 3D). For example, the 5′ UTR length of *rpoS*, which is one of the orthologous genes, was 566 in *E. coli*, while the length of the *K. pneumoniae rpoS* was 670. To investigate the sequence conservation between those comparable 5′ UTR regions, sequences of 5′ UTR regions from two species were aligned and percentage sequence identity for each 5′ UTR pair was calculated. The sequence variation of the 5′ UTR region along with the percentage identity of corresponding promoter and ORF is shown in Fig. 3E. Consequently, ORF sequence was found to be the most conserved element, followed by sequence of promoter regions and sequence of the 5′ UTR region as the most diverse regulatory element among them. The averages of sequence identity of orthologous ORFs, comparable conserved promoters, and their 5′ UTR were 88.9%, 79.0%, and 66.0%, respectively. In order to calculate the level of conservation of the regions surrounding translation start site of orthologous genes, sequences of 200 bp long regions around translation start sites were aligned for orthologous genes having clearly aligned translation start sites between *E. coli* and *K. pneumoniae* (Fig. 3F). In the 5′ UTR, there was a relatively more conserved regions 6 bp upstream of the translation start site. This region was considered to be the Shine-Dalgarno sequence of the ribosome binding site because in both species the most dominant distance between the Shine-Dalgarno sequence motif and translation start site was 6 nucleotides. In the coding region, the first codon, frequently ATG, was most conserved with slightly less conservation of the first nucleotide of the first codon. This was because the start codon, ATG, was replaced with GTG or TTG in some orthologs. In agreement with the wobble theory [32], the third nucleotide of each codon was least conserved. Interestingly, however, the second nucleotide was more conserved than the first in every codon analyzed. This might be because conservation of the second nucleotide can contribute to preserving the same amino acids like leucine, or amino acids with a similar property. Accordingly, codon analysis of the coding sequence between orthologous genes of the two species suggested that the majority of substitutions in the first nucleotide of the codon resulted in either keeping leucine or changing amino acids having similar properties, such as leucine/isoleucine, leucine/valine, valine/isoleucine, serine/threonine, glutamine/glutamic acid, or asparagine/aspartic acid.

In addition to species-specific gene content, *E. coli* and *K. pneumoniae* also exhibited differences in the organization of regulatory regions upstream of conserved orthologous genes. Different usage of TSSs and their promoter regions can contribute to varied regulation of genes downstream of those promoters, resulting in transcripts with different 5′ UTR. Moreover, both species extensively use multiple TSSs, which increase the complexity and diverse nature of regulatory regions. Thus, *E. coli* and *K. pneumoniae*, which are closely related, have regulatory regions of orthologous genes organized in a different manner.

### Comparison of regulatory non-coding small RNAs

The investigation of regulatory features of coding genes based on genome-wide TSSs and their comparison between two closely related enterobacteria showed that the two species share almost identical regulatory features. However, they deploy those regulatory features upstream of conserved or orthologous coding genes in a different manner, suggesting a variation of transcriptional regulation by using multiple TSSs and post-transcriptional regulation by having different 5′ UTRs, generated from a different set of TSSs. Since small regulatory RNAs can function in post-transcriptional control of gene expression in many processes including stress responses, metabolic reactions, and pathogenesis [33], [34], and identification of sRNAs in *K. pneumoniae* resulted in 34 orthologous sRNA pairs between two species, we compared sequences of those conserved sRNAs and investigated whether they would regulate their target genes in the same manner. This was done, similarly in previous studies [23], [35], [36].

**Figure 4:**
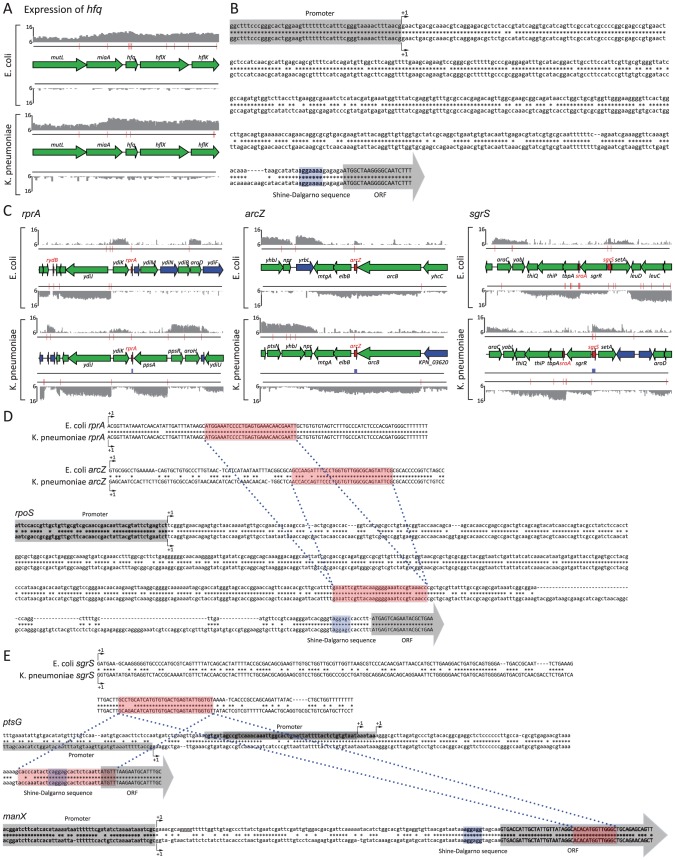
Comparison analysis of orthologous sRNAs. (A) Expression of RNA-binding protein *hfq* (B) Sequence conservation of regulatory region upstream of *hfq* ORF, including promoter, TSS and 5′ UTR. (C) Conservation and expression of non-coding regulatory sRNAs, *rprA*, *arcZ* and *sgrS*. (D) Sequence comparison analysis of *rprA* and *arcZ* regulating translation of *rpoS*. (E) Sequence comparison analysis of *sgrS* regulating translation of *ptsG* and *manX*.

The conserved RNA-binding protein Hfq, first discovered in *E. coli*, is a pleiotropic regulator that modulates the stability or the translation of an increasing number of mRNAs [37], [38]. Thus, knowledge of *hfq* in *K. pneumoniae* is preliminary in terms of analyzing and comparing sRNAs between two species. Similar to *E. coli* and other *K. pneumoniae* strains [39], [40], *hfq* of *K. pneumoniae* MGH78578 (KPN_04570), existed between conserved *miaA* and *hflX* in the genome. *E. coli* K-12 MG1655 *hfq* (b4172) and *K. pneumoniae* MGH78578 *hfq* (KPN_04570) had one TSS detected upstream of *hfq* and in the coding region of *miaA*, with the genomic position of 4,397,824 and 5,000,510, respectively (Fig. 4A). Similar to the high level of sequence conservation of the *hfq* ORF, sequences of promoter regions defined by experimental TSSs were perfectly conserved. 5′ UTR sequences were also highly conserved; preserving sequences for the Shine-Dalgarno sequence of the ribosome binding site 6 bp upstream of translation start sites in both species (Fig. 4B). This result supports the existence of a sequence of *K. pneumoniae hfq* ORF in the genome and is expressed with matching TSSs. Furthermore, sequence conservation of the promoter region and 5′ UTR indicates they could be regulated in a similar way, at least during the experimented condition.

With the occurrence of *hfq* in both species, we further investigated expression of orthologous sRNAs, compared their sequence, and analyzed possible working mechanisms in *K. pneumoniae* with prior knowledge of those sRNAs from *E. coli*. 34 orthologous sRNA candidates were confirmed to be expressed during exponential phase by TSS signals matching their 5′ ends. Their expression was also supported by expression profiling data (Figure S1 and Figure S2). However, those 34 expressed orthologous sRNAs showed different degrees of sequence conservation levels, ranging from 47.3% to 98.8% with an average of 83.1%. *rybB* has the most conserved sequence, whereas *sroH* has the least. Essentially, no sRNAs of *K. pneumoniae* had perfect sequence conservation compared to those of *E. coli*, which raised the question as to whether *K. pneumoniae* sRNA would work in a similar way as the *E. coli* sRNA. Thus, we compiled known target sites of *E. coli* sRNAs from the EcoCyc database [41], and mapped them onto the corresponding genomic sequence of *K. pneumoniae* (Table S6, Fig. 4D, Fig. 4E).

3 sRNAs, *rprA*, *arcZ*, and *dsrA* were known to regulate the expression of *rpoS* by making base paring in the middle region of 5′ UTR of *rpoS* mRNA with the aid of Hfq protein [42], [43], [44]. *rprA* and *arcZ* in *E. coli* target and bind to the same region of 5′ UTR of *rpoS*. Thus, based on the fact that those two sRNAs are expressed in *K. pneumoniae*, if the sequence of the target site in *rpoS* mRNA and the sequence of the corresponding region of sRNA which binds to that target site are conserved, then one can hypothesize that *rprA* and *arcZ* sRNAs of *K. pneumoniae* would regulate the expression of *rpoS* in a similar manner as in *E. coli*. As expected, *rprA* and *arcZ* of *K. pneumoniae* were expressed during exponential growth with TSSs, which match the TSSs of *E. coli*'s *rprA* and *arcZ* (Fig. 4C). Furthermore, regions that bind to the target site of *rpoS* were also conserved (Fig. 4D). Analogously, *rpoS* of *E. coli* and *K. pneumoniae* was expressed with TSS at 2,866,139 in *E. coli* and 3,401,901 in *K. pneumoniae*, and the sequence of the promoter region of *rpoS* was highly conserved between the two species, although the 5′ UTR defined by those TSSs showed significantly different lengths with a long nucleotide addition in the 5′ UTR of *K. pneumoniae rpoS*. However, the sequence of the target site of *rprA* and *arcZ* was almost perfectly conserved with one nucleotide replacement (Fig. 4D). Considering that *rpoS* was expressed from conserved promoters in both species, and *rprA* and *arcZ* targeting the conserved regions of the 5′ UTR of *rpoS* transcript were also conserved and expressed, it is quite likely those sRNAs in *K. pneumoniae* regulate the expression of *rpoS* in the same manner as in *E. coli*.

Furthering the analysis of *rpoS*-targeting *rprA* and *arcZ*, another sRNA *sgrS* was similarly analyzed. In *E. coli*, *sgrS* was shown to regulate expression of two metabolic transporters, *ptsG* and *manX*, by base-pairing dependent manner [45], [46]. Although *sgrS* sRNA of *E. coli* and *K. pneumoniae* was expressed during exponential phase (Fig. 4C), the sequence conservation was quite low at 56.3%. *ptsG* and *manX*, which are targeted and regulated by *sgrS* in *E. coli*, were also expressed in *K. pneumoniae*. However, *E. coli* and *K. pneumoniae ptsG* was expressed by different promoters, resulting in *ptsG* transcripts with different 5′ UTR, while *manX* was expressed by a conserved promoter with matching TSSs. Although the overall conservation level of *sgrS* between *E. coli* and *K. pneumoniae* was quite low, the region, which is known to bind to *ptsG* and *manX* in *E. coli*, was highly conserved (Fig. 4E). Besides its target sites in *ptsG* and *manX* transcripts were also highly conserved, which suggests *sgrS* would regulate the expression of *ptsG* and *manX* in a similar way as in *E. coli*.

Comparisons of sRNAs and their working mechanisms should be performed not only with just the sequence of sRNAs and their target sites, but also with the working context, including expression of those sRNAs, transcripts of genes containing target sites, and occurrence of Hfq, if the sRNA requires the protein. In depth comparisons of sRNAs and their working context suggest that many of the orthologous sRNAs identified with the TSS dataset of this study could work in a similar way as in *E. coli* since target sequences of those sRNAs and sequences of sRNAs known to bind to the target sites are conserved and exist in the primary transcript of target genes under the given condition. Moreover, high conservation of regions that bind to the target sites despite poor conservation of whole sRNA sequences suggests that sequence comparison and conservation can determine which region may be more important in terms of regulation and their working context.

## Discussion

*E. coli* K-12 MG1655 is an extensively studied laboratory strain with a wealth of genome-wide studies. As such, genome-wide TSS determination of the *E. coli* genome with single base pair resolution by deep sequencing has been performed by a number of studies [14], [15], [16]. However, *K. pneumoniae* has only recently been studied using genome-wide approaches [3]. A number of TSSs have been reported and investigated with specific focus on genes involved mostly in virulence [47], [48], [49], [50], [51], [52], [53], [54] and nitrogen metabolism [55], [56], [57], [58], [59], [60], [61], [62], [63], [64], [65] in other strains of *K. pneumoniae*. In addition to previously known TSSs of *K. pneumoniae*, this study extended the scope of knowledge by adding over 3,000 experimental TSSs for that species and by performing an in-depth look at the intergenic region of the *K. pneumoniae* genome.

Regulatory features discussed herein with *E. coli* and *K. pneumoniae* are not limited to the class of gammaproteobacteria. *G. sulfurreducens* in deltaproteobacteria [18], *H. pylori* in epsilonproteobacteria [17], *C. crescentus* in alphaproteobacteria [12] and methanogenic archaea *Methanosarcina mazei* [19] were also shown to have a significant amount of multiple TSS usage. Similarly, a mammalian promoter was also reported to have multiple TSSs [66], [67]. Thus, extensive use of multiple TSSs is a common strategy in a wide range of living organisms, exploiting alternative transcripts and providing complexity in gene expression and regulation. The level of multiple TSS usage differs by organism, however. *E. coli*, a generalist which can adjust and live in a wider range of environments, showed extensive usage of multiple TSSs, suggesting more complicated transcription regulation. On the other hand, *G. sulfurreducens* or *M. mazei*, a specialist that thrives in a more specific niche, showed lesser multiple TSSs. A significant fraction of operons had multiple TSSs in both *E. coli* and *K. pneumoniae* and encoded genes with essential functions, e.g., genes involved in amino acid biosynthesis, central metabolism, and transport, similar to *G. sulfurreducens* [18]. In addition to the usage of multiple TSSs, bacterial strains in different classes of proteobacteria show a similar distribution of 5′ UTR length. Like *E. coli* and *K. pneumoniae*, the preferred length of the 5′ UTR of *H. pylori* and *G. sulfurreducens* is 20–40 nucleotides in length. A distinctive regulatory function of the 5′ UTR was reported in yeast [68], however no correlation between the 5′ UTR length and function was found in *E. coli* or *K. pneumoniae*. However, when we compared the distribution of the 5′ UTR length between *E. coli* and *K. pneumoniae* belonging to the same COG functional group, most of the COG groups showed similar preferences for 5′ UTR length (Figure S5). Only the “Transcription” group showed significant differences (p-value of Wilcoxon rank sum test was 1.76×10^−5^).

Multiple promoters upstream of a gene can be regulated by transcription factors in different ways. For example, *rpoD* of *E. coli* which encodes sigma factor σ^70^ has multiple TSSs, and each promoter is recognized by σ^70^, σ^32^, or σ^24^, enabling expression of *rpoD* under conditions including exponential growth, heat shock, or other stresses [69], [70], [71]. Other transcription factors also contribute to the increasing complexity of promoter region structure. Transcription of an essential cell division protein operon of *E. coli*, *ftsQAZ*, is under the control of the two core promoters with two TSSs which are separated by 125 bp. Binding of the quorum sensing regulator, SdiA, activates the distal core promoter while it represses the proximal one [72]. *K. pneumoniae* also has that conserved operon *ftsQAZ*, and the TSSs of that particular operon were observed in both species and a similar regulation on expression of the *ftsQAZ* operon may be happening in *K. pneumoniae*. Another example is the *ure* operon (*ureDABCEFG*) in *K. pneumoniae*, which has two core promoters with distinct TSSs. One core promoter is NAC (nitrogen assimilation control protein) dependent, and the other is not [57]. For this operon, one TSS at the genomic position of 3,790,095 was identified during the mid-exponential growth in *K. pneumoniae* from this study. Thus, two closely related species in gammaproteobacteria, *E. coli* and *K. pneumoniae*, showed extensive usage of multiple TSSs, however they exhibited diverse organization of the regulatory region with different sets of promoters and associated TSSs. In addition to the presence of species-specific genes, this usage of multiple TSSs could potentially confer divergent regulation of orthologous genes, thereby contributing to phenotypic differences between two closely related species

Another advantage of having multiple TSSs is a transcript from each TSS has the different 5′ UTR upstream of coding region. Comparative analysis of 5′ UTRs between two species may provide insight into understanding similar or different roles of the 5′ UTR in the regulation of gene expression. One good example is a comparison of orthologous sRNAs and their binding onto the 5′ UTR region of target genes. Many orthologous sRNAs, including *rprA*, *arcZ*, and *sgrS*, showed enough evidence to postulate that their regulatory mechanism by the base pair dependent manner proven in *E. coli* may work similarly in *K. pneumoniae*. This conclusion is further supported by phylogenetic analysis of sRNA evolution in *E. coli* and *Shigella* genomes [73]. In the previous study, it is claimed that core or conserved sRNAs are more tightly integrated into cellular genetic regulatory networks, and over 80% of genes targeted by Hfq-associated core sRNAs have been transferred intact. 90% of orthologous sRNAs identified in our study were also categorized as core or conserved sRNAs in [73], supporting conserved regulatory mechanisms of those orthologous sRNAs.

An interesting additional attribute of the 5′ UTR sequence is that it can potentially serve as a transcription factor binding site, thereby contributing to the transcriptional regulation of a downstream gene or operon. For example, the ArgR (b3237) transcription regulator is known to bind to the promoter region and 5′ UTR of *argC* in *E. coli* [74], [75] (Figure S6A). The conserved promoter regions and TSSs were identified from the analysis of this study, and the sequence alignment of the upstream regulatory region and ORF suggests the binding regions of ArgR upstream of *argC* were highly conserved. Similarly, OxyR (b3961) transcription regulator, which auto-regulates its transcript by binding the promoter region and upstream regulatory region [76], also had conserved promoter regions and 5′ UTR defined by experimental TSSs identified in this study (Figure S6B). The binding regions of OxyR upstream of *oxyR* were also highly conserved. Considering those two transcription regulators, ArgR and OxyR, had a high level of amino acid sequence similarity of 94.2% and 96.1% respectively, and their target genes were expressed with conserved promoters and matching TSSs, it is likely that *K. pneumoniae*, ArgR, and OxyR may regulate *argC* and *oxyR* in a similar manner as in *E. coli*. However, unlike ArgR and OxyR regulation, NarL and AlaS transcription regulators showed opposite tendencies. In *E. coli*, NarL (b1221) regulates *ogt* by binding the upstream region of *ogt* [77] (Figure S6C). AlaS (b2697) of *E. coli* auto-regulates transcription of *alaS* by binding to the region covering parts of promoter region and the 5′ UTR [78] (Figure S6D). A high conservation level of those transcription factors (94.9% amino acid sequence similarity for NarL and 91.1% for AlaS) suggests the sequence motifs of their binding sites would be similar between *E. coli* and *K. pneumoniae*. However, sequence alignments of upstream regulatory regions of *ogt* and *alaS* between two species showed NarL and AlaS of *K. pneumoniae* may not regulate *ogt* and *alaS* by binding the same regions of binding sites. Thus, comparative analysis of upstream regions including the promoter region and the 5′ UTR of two closely related species can hint that the possibility of regulatory mechanisms of a lesser-studied microorganism, *K. pneumoniae*, by transferring ample knowledge from a well-studied microorganism such as *E. coli*.

Analysis of upstream regulatory regions performed in this study was based on the assumption that the current gene annotation of analyzed species is correct. Unlike the current gene annotation of *E. coli*, which is the most well-studied microorganism, the current gene annotation of *K. pneumoniae* was built by the computational methods and has not been fully confirmed with proteomic data, leaving the possibility of incorrect annotation of protein coding genes. Analyzing the sequence of coding regions and upstream regions of orthologous coding genes by sequence alignment suggested that many *K. pneumoniae* orthologous genes were longer than *E. coli* orthologous genes. The longer length of *K. pneumoniae* genes was mostly due to the fact that the current annotation of those genes had longer sequences at the N-terminus side (Figure S4A). When the genomic position of translation start sites were changed based on sequence alignment analysis of the coding region and upstream flanking region, 8 TSSs were found upstream of those changed translation start sites of *K. pneumoniae* orthologous coding genes (Table S7, Figure S4B).

TSS profiling through high-throughput sequencing techniques provides a comprehensive source of experimentally derived information related to the initiation of transcription. Annotation of non-coding regions in bacterial genomes, including the promoter regions and untranslated regions of transcripts, allows for the comparison of regulatory elements of transcription and translation between closely related species, and the identification of a spectrum from highly conserved to diverse regulatory elements. Direct comparison between cross species of bacteria also assists in transferring regulatory information of lesser-studied bacterial species and significantly improves annotation of regulatory regions. Thus, the comparative approach of this study provides a starting point for the determination of conserved and specific features of the transcriptional output of closely related bacteria at single nucleotide resolution.

## Materials and Methods

### Bacterial strains, media, and growth conditions

*Escherichia coli* K-12 MG1655 and *Klebsiella pneumoniae subsp. pneumoniae* MGH78578 were grown in glucose (2 g/L) minimal M9 medium containing 2 ml/L 1 M MgSO_4_, 50 µl/L 1 M CaCl_2_, 12.8 g/L Na_2_HPO_4_.7H_2_O, 3 g/L KH_2_PO_4_, 0.5 g/L NaCl, 1 g/L NH_4_Cl and 1 ml trace element solution (100×) containing 1 g EDTA, 29 mg ZnSO_4_.7H_2_O, 198 mg MnCl_2_.4H_2_O, 254 mg CoCl_2_.6H_2_O, 13.4 mg CuCl_2_, and 147 mg CaCl_2_. Glycerol stocks of the *E. coli* and *K. pneumoniae* strains were inoculated into the minimal medium supplemented with glucose and cultured at 37°C with constant agitation overnight. The cultures were diluted 1∶100 into 50 mL of the fresh minimal medium and then cultured at 37°C to an appropriate cell density.

### Total RNA isolation

Three milliliters of cells from mid-log (OD = 0.6) phase culture were mixed with 6 ml RNAprotect Bacteria Reagent (Qiagen). Samples were mixed immediately by vortexing for 5 seconds, incubated for 5 minutes at room temperature, and then centrifuged at 5000×*g* for 10 minutes. The supernatant was decanted and any residual supernatant was removed by inverting the tube once onto a paper towel. Total RNA samples were then isolated using RNeasy Plus Mini kit (Qiagen) in accordance with the manufacturer's instruction. Samples were then quantified using a NanoDrop 1000 spectrophotometer (Thermo Scientific) and quality of the isolated RNA was checked by visualization on agarose gels and by measuring the sample's A_260_/A_280_ ratio (>1.8).

### Modified 5′RACE for 5′tri-phosphorylated mRNA profiling

TSS determination protocol previously described [18] was adapted for the bacteria strains in the current study. To enrich intact 5′ tri-phosphorylated mRNAs from the total RNA, 5′ mono-phosphorylated ribosomal RNA (rRNA) and any degraded mRNA were removed by treatment with a Terminator 5′-Phosphate Dependent Exonuclease (Epicentre) at 30°C for 1 hr. The reaction mixture consisted of 10 µg purified total RNA, 1 µL terminator exonuclease, reaction buffer and RNase-free water up to total 20 µL. The reaction was terminated by adding 1 µL of 100 mM EDTA (pH 8.0). Intact tri-phosphorylated RNAs were precipitated by adding 1/10 volume of 3 M sodium acetate (pH 5.2), 3 volume of ethanol and 2 µL of 20 mg/mL glycogen. RNA was precipitated at −80°C for 20 min and pelleted, washed with 70% ethanol, dried in Speed-Vac for 7 minutes without heat and resuspended in 20 µL nuclease free water. The tri-phosphorylated RNA was then treated with RNA 5′-Polyphosphatase (Epicentre) to generate 5′-end mono-phosphorylated RNA for ligation to adaptors. The RNA sample from the previous step was mixed with 2 µL 10× reaction buffer, 0.5 µL SUPERase-In (Ambion), 1 µL RNA 5′-Polyphosphatase and RNase-free water up to 20 µL. The mixture was incubated at 37°C for 30 minutes and reaction was stopped by phenol-chloroform extraction. Ethanol precipitation was carried out for isolating the RNA as described above. To ligate 5′ small RNA adaptor (5′ GUUCAGAGUUCUACAGUCCGACGAUC 3′) to the 5′-end of the mono-phosphorylated RNA, the enriched RNA samples were incubated with 100 µM of the adaptor and 2.5 U of T4 RNA ligase (New England Biolabs). cDNAs were synthesized using the adaptor-ligated mRNAs as template using a modified small RNA RT primer from Illumina (5′ CAAGCAGAA GACGGCATACGANNNNNNNNN 3′′) and Superscript II Reverse Transcriptase (Invitrogen). The RNA was mixed with 25 µM modified small RNA RT primer and incubated at 70°C for 10 min and then at 25°C for 10 min. Reverse transcription was carried out at 25°C for 10 min, 37°C for 60 min, 42°C for 60 min and followed by incubation at 70°C for 10 min. A reaction mixture for reverse transcription consisted of the following components: 5×1^st^ strand buffer; 0.01 M DTT; 10 mM dNTP mix; 30 U SUPERase•In™ (Ambion); and 1500 U SuperScript™ II (Invitrogen). After the reaction, RNA was hydrolysed by adding 20 µL of 1 N NaOH and incubation at 65°C for 30 min. The reaction mixture was neutralized by adding 20 µL of 1 N HCl. The cDNA samples were amplified using a mixture of 1 µL of the cDNA, 10 µL of Phusion HF buffer (NEB), 1 µL of dNTPs (10 mM), 1 µL SYBR green (Qiagen), 0.5 µL of HotStart Phusion (NEB), and 5 pmole of small RNA PCR primer mix. The amplification primers used were 5′AATGATACGGCGACCACCGACAGGTTCAGAGTTCTACAGTCCGA3′ and 5′ CAA GCA GAA GAC GGC ATA CGA 3′. The PCR mixture was denatured at 98°C for 30 s and cycled to 98°C for 10 s, 57°C for 20 s and 72°C for 20 s. Amplification was monitored by a LightCycler (Bio-Rad) and stopped at the beginning of the saturation point. Amplified DNA was run on a 6% TBE gel (Invitrogen) by electrophoresis and DNA of size ranging from 100 to 300 bp were size fractionated. Gel slices were dissolved in two volumes of EB buffer (Qiagen) and 1/10 volume of 3 M sodium acetate (pH 5.2). The amplified DNA was ethanol-precipitated and resuspended in 15 µL DNase-free water (USB). The final samples were then quantified using a NanoDrop 1000 spectrophotometer (Thermo Scientific).

### Sequencing, data processing, and mapping

The amplified cDNA libraries from two biological replicates for each *E. coli* and *K. pneumoniae* were sequenced on an Illumina Genome Analyzer. Sequence reads for cDNA libraries for *E. coli* and *K. pneumoniae* were aligned onto the *E. coli* K-12 MG1655 genome (NC_000913) and *K. pneumoniae subsp. pneumoniae* MGH78578 genome with 5 plasmids (NC_009648, NC_009649, NC_009650, NC_009651, NC_009652, NC_009653), respectively, using Mosaik (http://code.google.com/p/mosaik-aligner) with the following arguments: hash size = 10, mismatach = 0, and alignment candidate threshold = 30 bp. Only reads that aligned to unique genomic location were retained. Two biological replicates were processed separately, and only sequence reads presented in both biological replicates were considered for further process. The genome coordinates of the 5′-end of these uniquely aligned reads were defined as potential TSSs. Among potential TSSs, only TSSs with the strongest signal within 10 bp window were kept to remove possible noise signals, and. TSSs with greater than or equal to 50% of the strongest signal upstream of an annotated gene were considered as multiple TSSs.

### Transcriptome analysis

Transcriptome dataset with oligonucleotide tiling microarrays for *E. coli* grown in glucose minimal media to the mid-exponential phase was taken from [16]. In order to get the transcriptome dataset for *K. pneumoniae*, the protocol previously described [18] was adapted for the *K. pneumoniae* in the current study. Briefly, 10 g of purified total RNA sample was reverse transcribed to cDNA with amino-allyl dUTP. The amino-allyl labeled cDNA samples were then coupled with Cy3 Monoreactive dyes (Amersham). Cy3 labeled cDNAs were fragmented to 50∼300 bp range with DNase I (Epicentre). High-density oligonucleotide tiling arrays consisting of 379,528 50-mer probes spaced 30 bp apart across the whole *K. pneumoniae* genome and 5 plasmids were used (Roche Nimblegen). Hybridization, wash and scan were performed in accordance with manufacturer's instruction. Two biological replicates were utilized for mid exponential growth under glucose minimal media. Probe level data were normalized with RMA (Robust Multiarray Analysis) algorithm [79] without background correction, as implemented in NimbleScan 2.4 software.

### Defining orthologous ORF in *E. coli* and *K. pneumoniae*

Genome annotation for *E. coli* K-12 MG1655 and *K. pneumoniae* MGH 78578 were obtained from the NCBI Genome Database. The refseq ID of *E. coli* K-12 MG1655 is NC_000913, and the refseq IDs of *K. pneumoniae* MGH 78578 genome and 5 plasmids are NC_009648, NC_009649, NC_009650, NC_009651, NC_009652 and NC_009653. In order to define orthologous ORFs between *E. coli* and *K. pneumoniae*, we performed reciprocal alignment for exhaustive pairs of amino acid sequences of ORFs in both strains, by using ClustalW2 software [80]. From the reciprocal alignment, we calculated percentage identity and percentage aligned scores, and used 50% as a cutoff for both percentage identity and percentage aligned scores of amino acid sequence alignment (Table S5).

### Data processing, visualization, and availability

Graphs representing the number of uniquely mapped reads per nucleotide were stored in GFF (generalized feature format) format files and visualized using MetaScope (http://sbrg.ucsd.edu/Downloads/MetaScope) and SignalMap software from Nimblegen (http://www.nimblegen.com/products/software/). Motif logos were calculated and drawn by MEME [27] and Venn diagrams and histograms were prepared by Microsoft Excel software. Experimental data were formatted to GFF format and visualized in MetaScope. The raw TSS reads for *E. coli* and *K. pneumoniae* and expression profiling dataset for *K. pneumoniae* have been deposited in the Gene Expression Omnibus (GEO) database (http://www.ncbi.nlm.nih.gov/geo/), GSE35822. Processed experimental data in this study are available at http://www.sbrg.ucsd.edu.

### Identification of potential sRNA

Potential sRNAs in *K. pneumoniae* were predicted by two methods. The first method is sRNA sequence search in the target region bound by neighboring orthologous genes. With the list of orthologous genes between *E. coli* and *K. pneumoniae*, closest orthologous genes neighboring each sRNA in *E. coli* were searched. Then, target region in *K. pneumoniae* genome was decided by neighboring orthologous genes. The sequence of *E. coli* sRNA was used to search conserved sequence in the target region in *K. pneumoniae* genome. For example, *E. coli glmY* is surrounded by *glrK* (b2556) and *purL* (b2557) orthologous genes. Thus, the target region in *K. pneumoniae* was chosen with the boundaries by *glrK* (KPN_02881) and *purl* (KPN_02882). Then, the sequence of *E. coli glmY* was searched in the target region, by sequence alignment between *glmY* and the target region with ClustalW2. This approach resulted in 48 putative sRNA candidates. The potential sRNA candidates were supplemented with prediction with Infernal (http://infernal.janelia.org) [25]. Rfam database 10.1 was used as model for sRNA prediction. Hits with E-value less than 10^−5^ were mapped to TSS dataset previously identified, and hits with the 5′ end matching to experimental TSSs were considered as potential sRNAs. The Infernal and Rfam approach resulted in 41 sRNA candidates. In sum, a total 50 number of sRNA candidates were prediction in a combination of two approaches.

### Categorization of promoter region based on conservation and presence of TSS

Each TSS experimentally identified was considered to be associated with one promoter region, so 50 bp long genomic region directly upstream of TSS was defined as promoter region. With the list of orthologous genes between *E. coli* and *K. pneumoniae*, TSS and its associated promoter region was categorized as species-specific promoter region (SSP), if that TSS was not assigned to any of orthologous genes. TSSs assigned to orthologous genes were categorized as one of three groups: conserved promoter region with TSS (CPT), conserved promoter region with no matching TSS (CPNT) or orphan promoter region (OP). For each orthologous gene, all TSSs assigned to that gene in both species were used to define promoter regions. Then the sequence of each promoter region of one species was aligned onto the sequence of 800 bp long genomic region upstream of the orthologous gene in the other species, in order to see the 3′ end of the alignment match with any TSS of the other species with 2 bp tolerance. If there is a matching TSS, the promoter region of that TSS was aligned again back onto the 800 bp upstream region of the first species, and if the 3′ end of the second alignment matched with the first TSS, then those two TSS in both species were categorized as CPT. If the 3′ end of the first alignment didn't match any TSS of the other species, then the sequence of alignment of the other species was aligned back onto the upstream region of the first species. If the 3′ end of the second alignment matched with the first TSS, then the promoter region defined by that TSS was categorized as CPNT. If the 3′ end of the second alignment did not match with the first TSS, then the promoter region was categorized as OP.

## Supporting Information

Figure S1 Schematic drawing of annotated TSSs assigned to orthologous sRNAs and their neighboring coding genes in *E. coli* and *K. pneumoniae* (The first half of 17 sRNAs).
(TIF)

Figure S2 Schematic drawing of annotated TSSs assigned to orthologous sRNAs and their neighboring coding genes in *E. coli* and *K. pneumoniae* (The other half of 17 sRNAs).
(TIF)

Figure S3 Schematic drawing of annotated TSSs assigned to non-orthologous sRNAs and identified cis-acting regulatory elements (A) Non-orthologous *K. pneumoniae* sRNAs: *rnai* and *ryhB-2* (B) identified cis-acting regulatory elements.
(EPS)

Figure S4 Comparative analysis on possible misannotation in the current annotation of *K. pneumoniae* (A) Comparison of the length difference between the whole orthologous ORFs and their N-terminus alignments. (B) Updated annotation of *ecnB* and *rcnR* by TSS.
(EPS)

Figure S5 Comparison of 5′ UTR length distribution between *E. coli* and *K. pneumoniae* by COG functional categories.
(EPS)

Figure S6 Comparison analysis of transcription factor binding sites upstream of orthologous genes (A) ArgR binding on *argC* (B) OxyR binding on *oxyR* (C) NarL binding on *ogt* (D) AlaS binding on *alaS.*
(EPS)

Table S1 Experimentally derived TSS annotation for *E. coli* and *K. pneumoniae*.
(XLSX)

Table S2 TSS of *E. coli* sRNAs identified by different experimental methods.
(XLSX)

Table S3 sRNAs and cis-acting regulatory elements in *K. pneumoniae*.
(XLSX)

Table S4 Leaderless mRNAs identified in *E. coli* and *K. pneumoniae*.
(XLSX)

Table S5 Orthologous ORFs between *E. coli* and *K. pneumoniae*.
(XLSX)

Table S6 Comparison analysis of sRNA target sites between *E. coli* and *K. pneumoniae*.
(XLSX)

Table S7 Updated translation start sites in *K. pneumoniae*.
(XLSX)
