# Supplementary figures and images for: Comparative Analysis of Regulatory Elements between Escherichia coli and Klebsiella pneumoniae by Genome-Wide Transcription Start Site Profiling
Source: PLoS Genet. 2012 Aug 9;8(8):e1002867. doi: 10.1371/journal.pgen.1002867 (PMC3415461; doi:10.1371/journal.pgen.1002867)

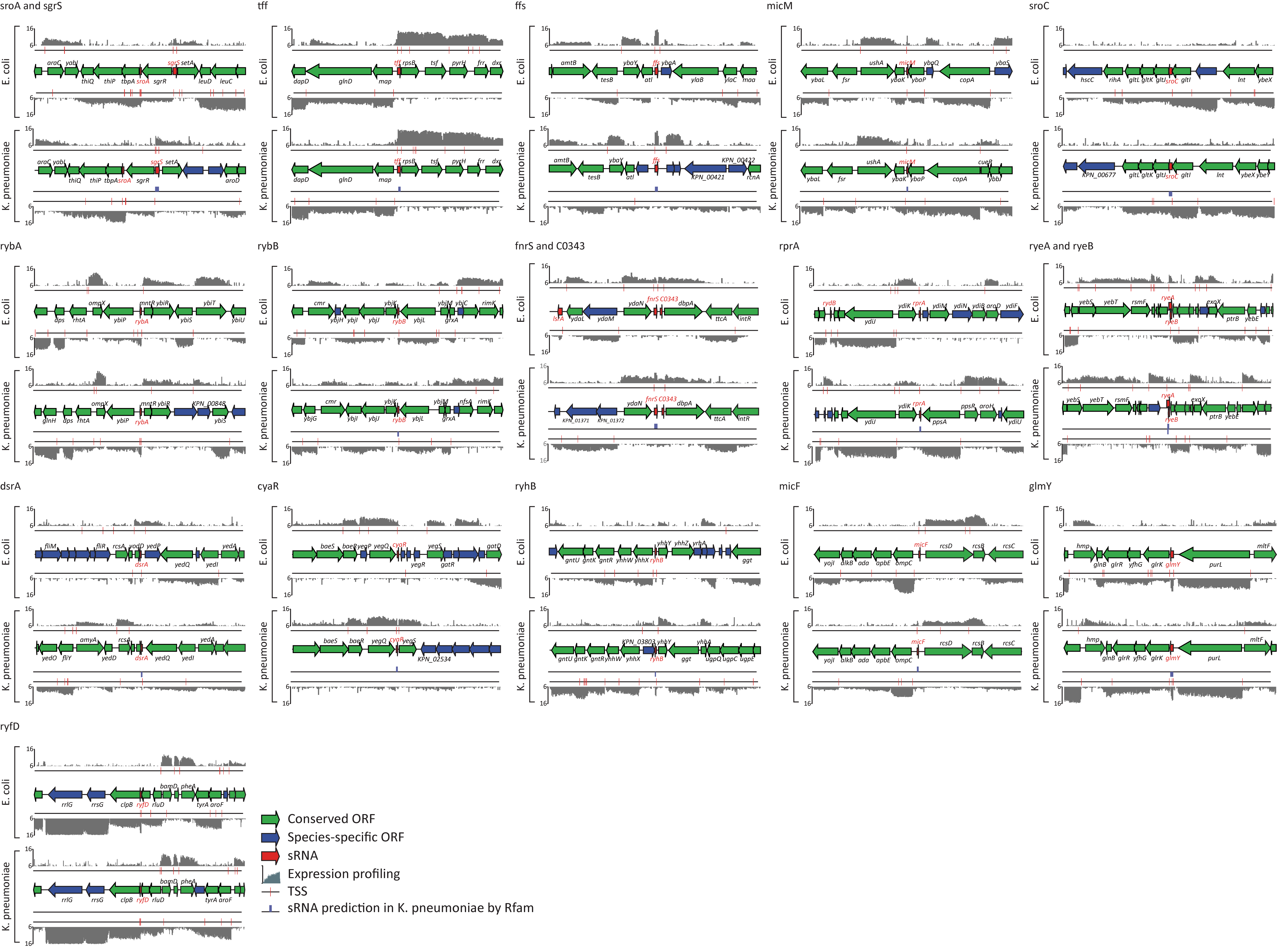

Supplement: Figure S1 — Schematic drawing of annotated TSSs assigned to orthologous sRNAs and their neighboring coding genes in E. coli and K. pneumoniae (The first half of 17 sRNAs). (TIF) [file pgen.1002867.s001.tif]

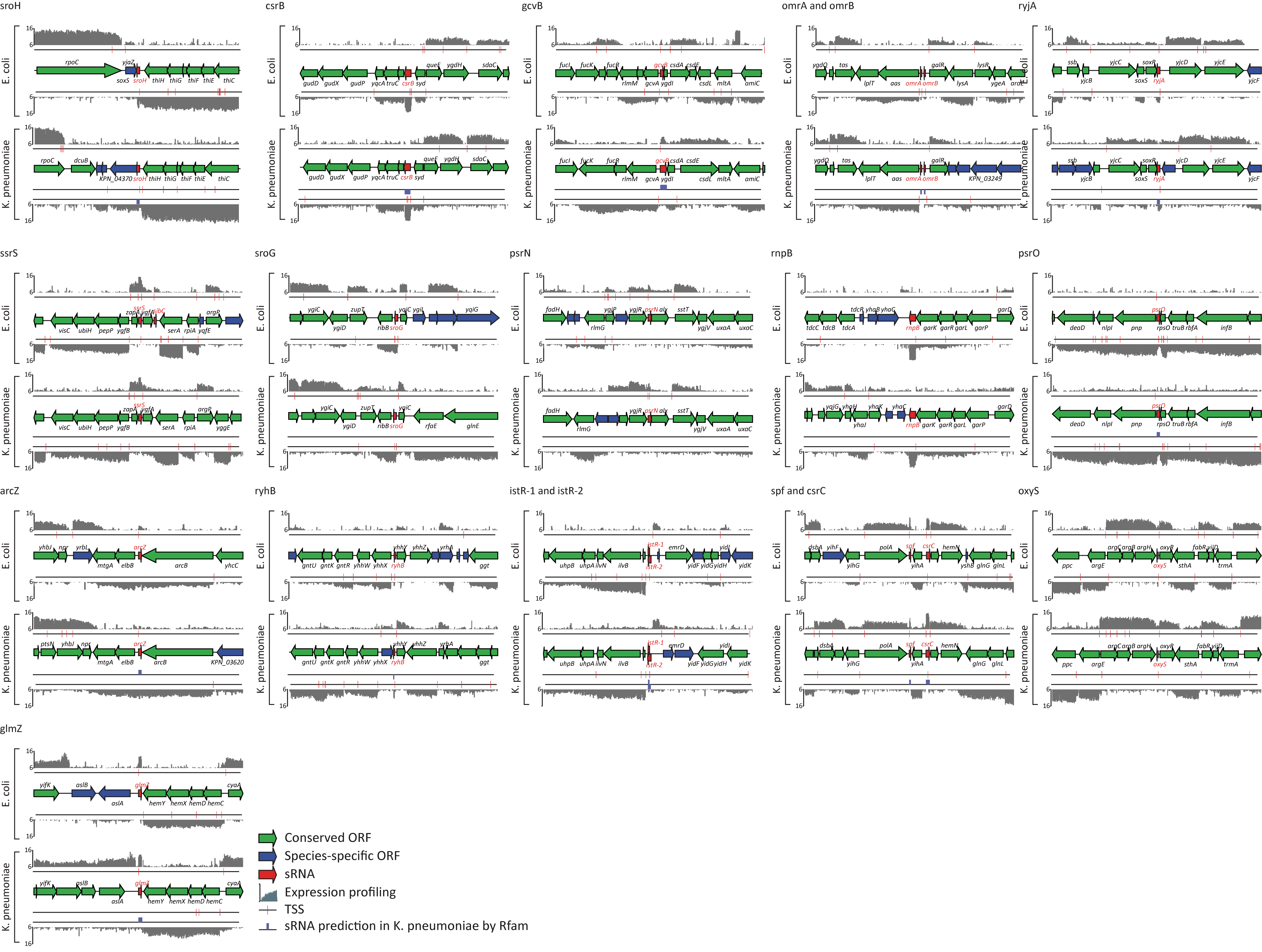

Supplement: Figure S2 — Schematic drawing of annotated TSSs assigned to orthologous sRNAs and their neighboring coding genes in E. coli and K. pneumoniae (The other half of 17 sRNAs). (TIF) [file pgen.1002867.s002.tif]
